# Supplementary material for: Splice-Junction-Based Mapping of Alternative Isoforms in the Human Proteome
Source: Cell Rep. Author manuscript; Available in PMC 2020 Jan 15. (PMC6961840; doi:10.1016/j.celrep.2019.11.026)
Supplement: 2 [file NIHMS1546469-supplement-2.zip › DF1/PXD010154/Thyroid/Thyroid_2_TG_SAIICGLLSSPSVLLCNVK.pdf]

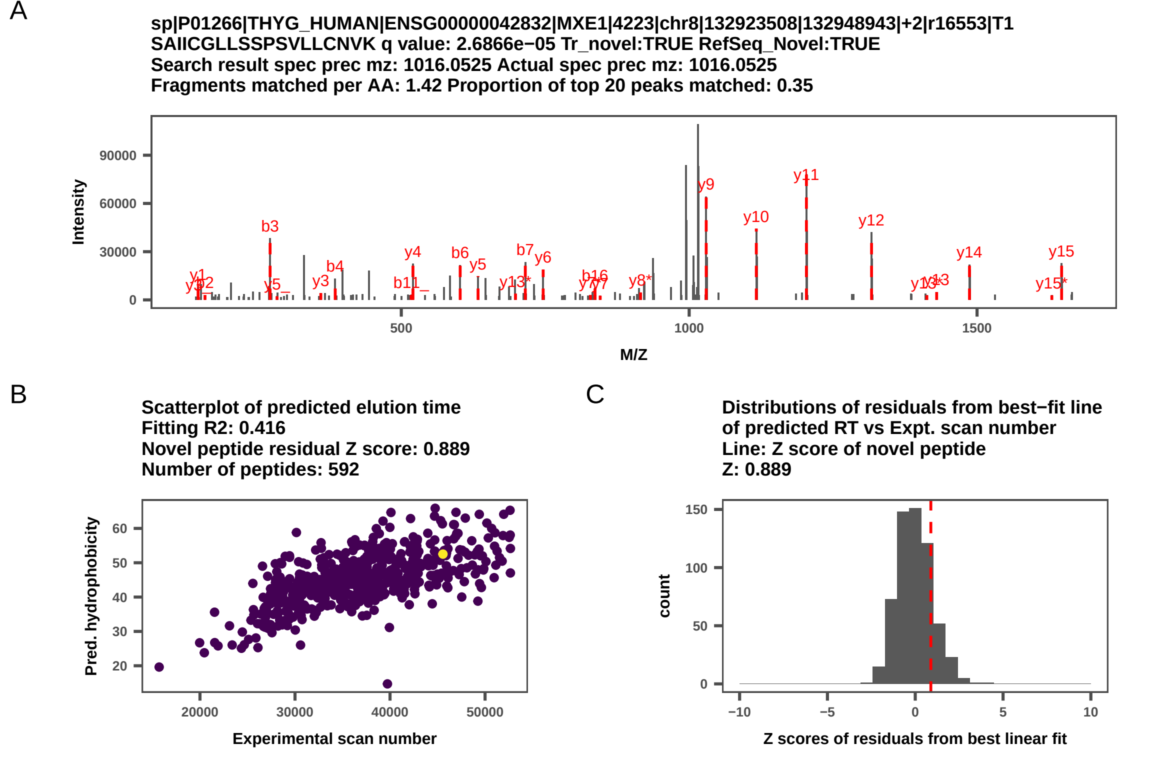
A
sp|P01266|THYG_HUMAN|ENSG00000042832|MXE1|4223|chr8|132923508|132948943|+2|r16553|T1
SAIICGLLSSPSVLLCNVK q value: 2.6866e−05 Tr_novel:TRUE RefSeq_Novel:TRUE
Search result spec prec mz: 1016.0525 Actual spec prec mz: 1016.0525
Fragments matched per AA: 1.42 Proportion of top 20 peaks matched: 0.35
Intensity
90000
60000
30000
0
500
1000
1500
M/Z
y1
b3
y5_
y3
b4
y4
b11_
b6
y5
b7
y6
y13*
b16
y8*
y9
y10
y11
y12
y14
y15
y15*
B
Scatterplot of predicted elution time
Fitting R2: 0.416
Novel peptide residual Z score: 0.889
Number of peptides: 592
Pred. hydrophobicity
60
50
40
30
20
20000
30000
40000
50000
Experimental scan number
C
Distributions of residuals from best−fit line
of predicted RT vs Expt. scan number
Line: Z score of novel peptide
Z: 0.889
count
150
100
50
0
−10
−5
0
5
10
Z scores of residuals from best linear fit
